# Correlation between bacterial G+C content, genome size and the G+C content of associated plasmids and bacteriophages

**DOI:** 10.1099/mgen.0.000168

**Published:** 2018-04-10

**Authors:** Apostolos Almpanis, Martin Swain, Derek Gatherer, Neil McEwan

**Affiliations:** ^1^​ Aberystwyth University, Aberystwyth, UK; ^2^​ Newcastle University, Newcastle-upon-Tyne, UK; ^3^​ Lancaster University, Lancaster, UK; ^4^​ School of Pharmacy and Life Sciences, Robert Gordon University, Aberdeen, UK

**Keywords:** genome G+C content, genome length, bacteria, plasmids

## Abstract

Based on complete bacterial genome sequence data, we demonstrate a correlation between bacterial chromosome length and the G+C content of the genome, with longer genomes having higher G+C contents. The correlation value decreases at shorter genome sizes, where there is a wider spread of G+C values. However, although significant (*P*<0.001), the correlation value (Pearson *R*=0.58) suggests that other factors also have a significant influence. A similar pattern was seen for plasmids; longer plasmids had higher G+C values, although the large number of shorter plasmids had a wide spread of G+C values. There was also a significant (*P*<0.0001) correlation between the G+C content of plasmids and the G+C content of their bacterial host. Conversely, the G+C content of bacteriophages tended to reduce with larger genome sizes, and although there was a correlation between host genome G+C content and that of the bacteriophage, it was not as strong as that seen between plasmids and their hosts.

## Data Summary

Jupyter notebooks for the analysis of the data can be found at: https://github.com/atolgrp/Microbial-G-C-Content.

**Impact Statement**

Larger genomes provide an opportunity for containing more genes due to the larger amount of DNA. However, the reasons associated with this are still debated and relatively unclear. Using genome sequences accessible from public databases, this paper examines the potential relationship between G+C content and genome length. In addition to studying bacterial genomes, the work also looks at this relationship between G+C content and genome length for both plasmids and bacteriophages. We also compare the G+C content of both plasmid and bacteriophage genomes relative to the G+C content of the organism from which they were isolated. In general, we found that larger bacterial genomes tend to have higher G+C contents, as was the case for plasmids. However, in bacteriophages, the G+C content appeared to reduce with an increase in size. There was a high level of correlation between the G+C content of plasmids and their host organism, a pattern that was seen to some extent between bacteriophages and the organisms they infect, but with a lower correlation level.

## Introduction

The redundancy of the genetic code, where as many as six different codons may encode a single amino acid, allows at least some tolerance of the nucleotides used by different organisms. This tolerance, at least in part, means that the bacterial genomic guanine+cytosine (G+C) content may vary enormously, depending on the species. Recently, this range was shown to extend from 17 to 75 mol% [1]. The factors influencing this variation have been debated for at least 50 years [2, 3], including the suggestion that mutational bias acts upon genomes. This bias, together with environmental factors, was thought to exert a selection pressure towards the most adapted genome composition for a given habitat. Subsequent research suggested that this mutational bias generally acts across all bacterial species and promotes a trend towards genomes with higher adenine+thymine (A+T) content [4–6]. Other research revealed that the G+C content of individual bacterial species is correlated to a number of factors. These factors are not mutually exclusive and have included variables such as the organism’s living environment [7], the ability or inability to fix atmospheric nitrogen [8], an organism’s preference for aerobic or anaerobic conditions [8, 9], and normal optimal temperature range [10, 11]. The interconnection of these intrinsic and extrinsic factors means that no single condition is likely to be responsible for the G+C content of an organism, but rather this is due to multiple factors, which in turn makes identification of the relationships between them difficult to analyse.

Various approaches have been adopted to analyse the factors that might influence the G+C content, including traditional (laboratory-based) microbiology and *in silico* analyses using phylogenetic studies in an attempt to identify similarities between organisms with particular G+C contents. One of the simplest hypothesized relationships was that of a potential correlation between the genomic G+C content of an organism and genome size. This was first proposed by Sueoka [3] and has been studied further by others since (e.g. [12–15]). Initial investigations relying on examining the genome size posed problems due to shearing of DNA during the extraction process, thereby potentially leading to under-estimations of the correct size. Even with the advent of pulsed-field gel electrophoresis [16], which greatly overcame the potential problem of DNA fragmentation, this issue was not fully resolved. However, with the improvements to DNA sequencing methods, particularly the increased use of next-generation sequencing to determine complete genome sequences, accurate values for both genome size and G+C content are becoming increasingly available.

The present study makes use of data from genome sequences and is, to our knowledge, the largest investigation undertaken to date to assess the potential relationship between genome size and G+C content. Furthermore, it also includes plasmids in the analysis and compares their G+C content to that of their host organism.

## Methods

Data were downloaded from the National Center for Biotechnology Information (NCBI) database, on 12 June 2017. For that purpose, Linux shell commands were used (awk for address parsing and wget for downloading), wrapped in a python script. At the time of downloading, the database contained 14 774 genome entries. The downloaded dataset included a number of draft and incomplete sequences. Only entries containing the text string ‘complete’ in their Fasta definition line (defline) were selected. The same criterion was applied for the separation of plasmids and phages, namely the existence of the text strings ‘plasmid’ and ‘phage’. The rest were assigned as bacterial genomic sequences. The majority of bacteriophage genomes were downloaded from a separate directory in NCBI, but some sequences were also included in the main dataset for microbial genomes. These two datasets were merged after cleaning and any duplicates were removed computationally. Further entries described in their defline as ‘putative’ or ‘endosymbiont’ were also removed. This subset was comparatively small and lacked clear annotation.

All data manipulation and statistical analysis was performed using python 2.7 (implemented in anaconda 2, v4.4.0) (Python Software Foundation, https://www.python.org), in a Linux 64-bit environment. Standard python libraries were used for data cleaning and subsequent analysis, such as *pandas*, *scipy* and *numpy.*

Ordinary least squares (OLS) was applied for linear regression, using python with *statsmodels.OLS*. This method still provides an unbiased regression estimation in the presence of unequal variance across the data (heteroskedasticity) [17], as the latter were evident across all datasets. One drawback, however, is that when heteroskedasticity is present, OLS has no predictive power, as the error margins and *P* values can be too small or too large, and cannot be trusted. To mitigate this effect, OLS was used with the HCCM (heteroskedasticity consistent covariance matrix) method [17], which in python *statsmodels* is implemented with the *cov_type=‘HC0’* option.

Plots were produced using *matplotlib* (v2.0.1) [18] and *seaborn* (v0.7.1) (M. Waskom, O. Botvinnik, D. O'Kane, P. Hobson, D. C. Gemperline *et al*., 2016). To enable researchers to easily re-apply our analysis protocols, we have made all code used to generate plots and tables available as jupyter notebooks at https://github.com/atolgrp/Microbial-G-C-Content.

## Results

After cleaning, the dataset comprised 12 424 complete genome sequences from bacterial sources; 6671 from bacterial chromosomes, 5744 from plasmids and 4580 from phages. Inevitably, extensively studied microbial species, such as *Escherichia coli* or *Bacillus* spp., were represented by more than one strain.

The G+C content ranged from 13.5 mol% (*Zinderia insecticola* CARI) to 87.5 mol% (*Streptomyces autolyticus* strain CGMCC0516 plasmid), with a mean value of 48.4 mol%. In distributions with heavy skew, the median is a better estimate of a representative value. For the whole dataset, this was slightly higher than the mean, at 48.5 mol%. Lengths varied from 744 bp (*Tremplaya phenacola* PAVE plasmid) to 16 Mb (*Minicystis rosea* strain DSM 24000). Mean and median lengths were 2.08 and 1.64 Mb, respectively.

### Bacterial genomes

Bacterial genomic sequence length ranged from 112 kb (*Nasuia deltocephalinicola* strain PUNC) to 16 Mb (*M. rosea* strain DSM 24000), with a mean length of 3.66 Mb and a median of 3.78 Mb (Table 1). The lowest G+C content was that of *Z. insecticola* CARI at 13.5 mol% and the highest that of *Anaeromyxobacter dehalogenans* 2CP-C, at 74.9 mol% (Table 2). The mean G+C content was 48.8 mol% and the median was 49.3 mol%.

**Table 1.** Microbes, plasmids and phages with extreme values of length The five longest and shortest values are shown in each case. G+C values have been rounded to one decimal place.

**Genome**		**Length (bp)**	**G+C (mol%)**
**Bacterial genomes**			
Longest bacterial genomes			
1	*Minicystis rosea* strain DSM 24000 (CP016211.1)	16 040 666	69.1
2	*Sorangium cellulosum* So0157-2 (CP003969.1)	14 782 125	72.1
3	*Nonomuraea* sp. ATCC 55076 (CP017717.1)	13 047 416	71.8
4	*Sorangium cellulosum* ‘So ce 56' (AM746676.1)	13 033 779	71.4
5	*Archangium gephyra* strain DSM 2261 (CP011509.1)	12 489 432	69.4
Shortest bacterial genomes			
1	*Candidatus Nasuia deltocephalinicola* strain PUNC (CP013211.1)	112 031	16.6
2	*Candidatus Nasuia deltocephalinicola* str. NAS-ALF (CP006059.1)	112 091	17.1
3	*Candidatus Hodgkinia cicadicola* isolate TETUND1 (CP007232.1)	133 698	46.8
4	*Candidatus Tremblaya princeps* PCIT (CP002244.1)	138 927	58.8
5	*Candidatus Tremblaya princeps* PCVAL (CP002918.1)	138 931	58.8
**Plasmid genomes**			
Longest plasmids			
1	*Cupriavidus metallidurans* CH34 megaplasmid (CP000353.2)	2 580 084	63.6
2	*Burkholderia caribensis* MBA4 plasmid (CP012748.1)	2 555 069	62.4
3	*Rhizobium gallicum bv. gallicum* R602 plasmid pRgalR602c (CP006880.1)	2 466 951	59.4
4	*Sinorhizobium fredii* NGR234 plasmid pNGR234b (CP000874.1)	2 430 033	62.3
5	*Rhizobium gallicum* strain IE4872 plasmid pRgalIE4872d (CP017105.1)	2 388 366	59.2
Shortest plasmids			
1	*Candidatus Tremblaya phenacola* PAVE plasmid (CP003983.1)	744	42.2
2	*Lactococcus lactis subsp. lactis* KLDS 4.0325 plasmid 2 (CP007042.1)	870	32.6
3	*Enterococcus faecium strain* ISMMS_VRE_1 plasmid ISMMS_VRE_p5 (CP012433.1)	886	31.3
4	*Borreliella garinii* strain CIP 103362 plasmid cp32 (CP018755.1)	1 085	30.4
5	*Acinetobacter baumannii* strain JBA13 plasmid pJBA13_2 (CP020583.1)	1 109	59.1
**Phage genomes**			
Longest phages			
1	*Agrobacterium* phage Atu_ph07 (MF403008.1)	490 380	37.1
2	*Salicola* phage SCTP-2 (MF360958.1)	440 001	30.0
3	*Pectobacterium* phage CBB (KU574722.1)	378 379	35.9
4	*Aureococcus anophagefferens* phage *BtV-01* (NC_024697.1)	370 920	28.7
5	*Cronobacter* phage vB_CsaM_GAP32 (JN882285.1)	358 663	35.6
Shortest phages			
1	*Leuconostoc* phage L5 (L06183.1)	2 435	33.3
2	*Enterobacteria* phage M (JX625144.1)	3 405	48.0
3	*Enterobacterio* phage KU1 (AF227250.1)	3 486	46.5
4	*Enterobacteria* phage C-1 INW-2012 (JX045649.1)	3 523	48.4
5	*Enterobacterio* phage MS2 isolate DL52 (JQ966307.1)	3 525	51.0

**Table 2.** Microbes, plasmids and phages with extreme values of G+C content Only the five highest and lowest values are shown in each case. G+C values have been rounded to one decimal place.

Genome		**Length (bp)**	**G+C (mol%)**
**Bacterial genomes**			
Organisms with highest bacterial genome G+C content			
1	*Anaeromyxobacter dehalogenans* 2CP-C (CP000251.1)	5 013 479	74.9
2	*Anaeromyxobacter* sp. K (NC_011145.1)	5 061 632	74.8
3	*Streptomyces rubrolavendulae* strain MJM4426 (CP017316.1)	6 543 262	74.8
4	*Corynebacterium sphenisci* DSM 44792 (NZ_CP009248.1)	2 594 799	74.7
5	*Cellulomonas fimi* ATCC 484 (NC_015514.1)	4 266 344	74.7
Organisms with lowest bacterial genome G+C content			
1	*Candidatus Zinderia insecticola* CARI (CP002161.1)	208 564	13.5
2	*Candidatus Carsonella ruddii* CE isolate Thao2000 (CP003541.1)	162 589	14.0
3	*Candidatus Carsonella ruddii* HC isolate Thao2000 (CP003543.1)	166 163	14.2
4	*Candidatus Carsonella ruddii* CS isolate Thao2000 (CP003542.1)	162 504	14.2
5	*Candidatus Carsonella ruddii* HT isolate Thao2000 (CP003544.1)	157 543	14.6
**Plasmid genomes**			
Plasmids with highest G+C content			
1	*Streptomyces autolyticus* CGMCC0516 plasmid unnamed3 (NZ_CP019460.1)	30 888	87.5
2	*Streptomyces autolyticus* CGMCC0516 plasmid unnamed8 (NZ_CP019465.1)	15 591	83.3
3	*Streptomyces cattleya* NRRL 8057 plasmid pSCAT (FQ859184.1)	1 809 491	73.3
4	*Streptomyces cattleya* DSM 46488 plasmid pSCATT (CP003229.1)	1 812 548	73.3
5	*Streptomyces* sp. FR-008 plasmid pSSFR2 (CP009804.1)	24 272	72.9
Plasmids with lowest G+C content			
1	*Candidatus Baumannia cicadellinicola* strain B-GSS plasmid (CP011788.1)	3 465	20.3
2	*Blattabacterium* sp. (*Nauphoeta cinerea*) plasmid (NC_022551.1)	3 674	20.6
3	*Borrelia burgdorferi* B31 plasmid lp21 (CP009673.1)	18 777	20.6
4	*Streptobacillus moniliformis* DSM 12112 plasmid pSMON01 (CP001780.1)	10 702	20.9
5	*Brachyspira intermedia* PWS/A plasmid pInt (CP002875.1)	3 260	21.0
**Phage genomes**			
Phage with highest G+C content			
1	*Streptomyces* phage SV1 (NC_018848.1)	37 612	72.7
2	*Streptomyces* phage PapayaSalad (KY092481.1)	38 411	72.6
3	*Streptomyces* phage Picard (KY092480.1)	39 522	72.6
4	*Streptomyces* phage Mojorita (KY092482.1)	38 496	72.5
5	*Streptomyces* phage ToastyFinz (KY676784.1)	39 693	72.5
Phage with lowest G+C content			
1	*Spiroplasma* phage SVTS2 (AF133242.2)	6 825	20.3
2	*Spiroplasma* phage 1-R8A2B (NC_001365.1)	8 273	22.9
3	*Spiroplasma* phage SVGII3 (AJ969242.1)	7 878	23.0
4	*Spiroplasma* phage 1-C74 (NC_003793.1)	7 768	23.2
5	*Mycoplasma* phage phiMFV1 (AY583236.1)	18 855	24.8

**Fig. 1.**
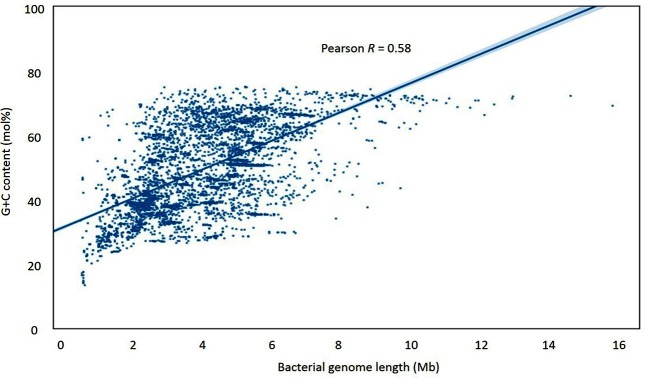
Scatterplot of G+C content versus sequence length for bacterial chromosomal sequences, showing an approximately triangular shape associated with their relation. Pearson’s *R* indicates that about 58 mol% of the G+C content variation can be explained by genome length, although there is also apparent heteroskedasticity. G+C content is plotted using values to the nearest percentage point.

The data showed a prominent heteroskedasticity. Longer sequences tended to have higher G+C content values, while variation in G+C started high in short genomes and decreased as genomes became longer. In keeping with previous research [13, 14], this creates a data plot of a roughly triangular shape (Fig. 1). There is a positive correlation between genomic G+C content and bacterial genome length, though this is not a simple one: length is associated more with the range of G+C content, rather with its absolute value. As noted above, small sequences accommodate the whole range of G+C content, while as length increases, G+C values tend to occupy the upper part of the range. This is in keeping with the data in Table 1, where the five longest genome sequences all have G+C values of 69 mol% or more, whilst the shortest five examples range from 16.6 to 58.8 mol%.

Therefore, trying to fit a linear regression model onto this dataset was potentially problematic. Using heteroscedasticity-robust regression, the linear model explained only a small proportion of the variation (Pearson *R*=0.58, *P*<0.001). This is equivalent to an *r*^2^ of 0.34 and, thus, around 66 mol% of the variation in G+C content cannot be accounted by this model. The heteroskedastic pattern could not be removed by log or root data manipulation, although the Pearson's *R* value for genomes was raised to 0.61 by log-log transformation (data not shown).

### Plasmid genomes

Generally, plasmids were much smaller in size, although a few larger examples existed at >500 kb, e.g. those found in bacteria belonging to the genus *Rhizobium*. Table 1 shows that the length ranged from 744 bp (*T. phenacola* PAVE plasmid) to 2.58 Mb (*Cupriavidus metallidurans* CH34 megaplasmid). The plasmid with the lowest G+C was from *Baumannia cicadellinicola* strain B-GSS at 20.3 mol%, whilst the two plasmids with the highest G+C content (87.5 and 83.3 mol%, respectively) were from the same organism: *S. autolyticus* strain CGMCC0516 (Table 2).

**Fig. 2.**
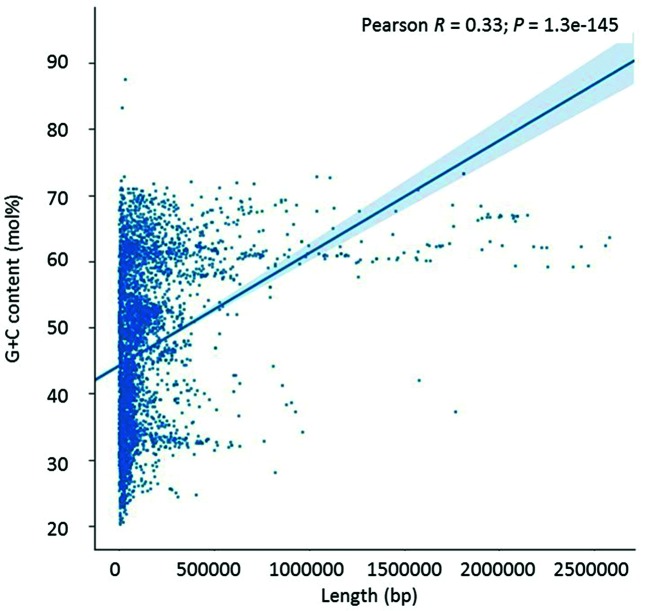
Scatterplot of plasmid G+C content versus plasmid sequence length, showing an approximately rotated L-shape. G+C content is shown to the nearest mol% value.

Plasmids showed a similar pattern of G+C content variation to that seen in bacterial genomes, namely high variability of G+C in smaller sequences and a tendency for high G+C content as the size increased (Fig. 2). However, given the generally smaller length of these plasmids, the general abundance of shorter sequence lengths generated a rotated L-shape pattern when plotted, rather than the triangular shape seen for bacterial chromosomes.

### Correlation between plasmid and host G+C content

**Fig. 3.**
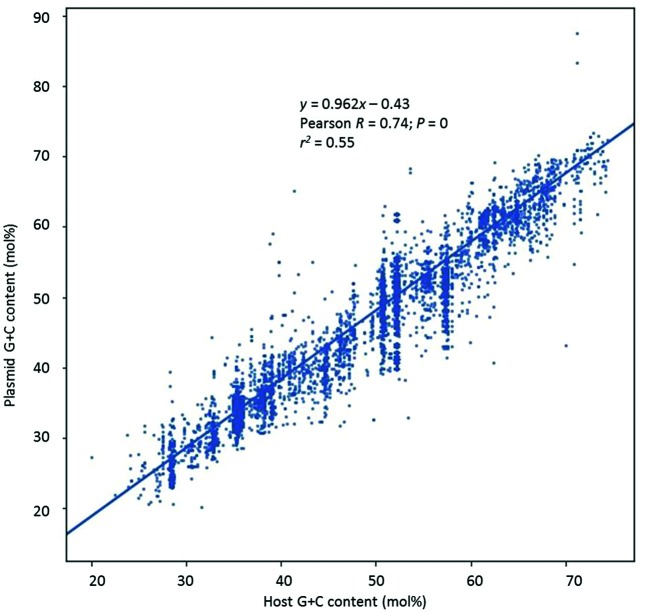
Comparison of the G+C content of plasmids versus that of their host. G+C content is shown to the nearest mol% value.

A linear relationship (Fig. 3) was evident between plasmid G+C content and the corresponding G+C content of the host organism (Pearson *R* value=0.74, *P*<0.0001), although the variance was again not consistent throughout. The linear equation obtained showed approximately a one-to-one relationship between the two variables, with the plasmid G+C content increasing about 0.96 mol% for every 1 mol% increase in host G+C. Nevertheless, about 45 mol% of the variation was not explained by this relationship (*r*^2^=0.55, *P*<0.0001).

### Phage genomes

Like plasmids, phages were generally small in size, although a few larger examples existed, the largest being from at almost 500 kb. Table 1 shows that the length ranged from 2435 bp (*Leuconostoc* phage L5) to 490 kb (*Agrobacterium* phage Atu_ph07). The phage with the lowest G+C was SVTS2 from *Spiroplasma* at 20.3 mol%, whilst the phage with the highest five G+C content values (72.7 to 72.5 mol%) were all from *S. autolyticus* (Table 2).

**Fig. 4.**
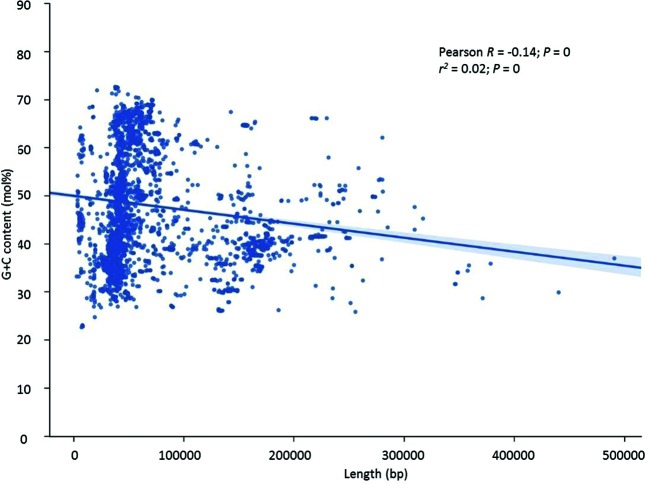
Scatterplot of phage G+C content versus phage sequence length, showing that longer phages tend to have a lower G+C content. G+C content is shown to the nearest mol% value.

Phages showed the pattern seen in both bacterial and plasmid genomes, namely high variability of G+C in smaller sequences. However, unlike bacterial and plasmid genomes, those with larger genomes showed a tendency for lower G+C content (Pearson *R* value=−0.14, *P*<0.0001) as the size increased (Fig. 4).

### Correlation between phage and host G+C content

**Fig. 5.**
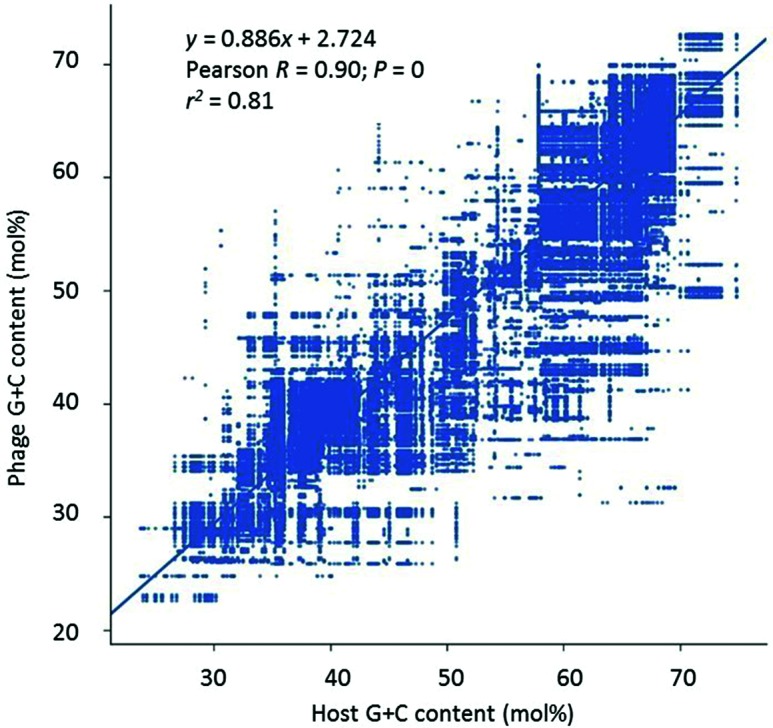
Comparison of the G+C content of phages versus that of their host. G+C content is shown to the nearest mol% value.

A linear relationship (Fig. 5) was evident between phage G+C content and the corresponding G+C content of the host organism (Pearson *R* value=0.90, *P*<0.0001). This was the best regression result for the whole dataset. The linear equation obtained approached a one-to-one relationship between the two variables, with the phage G+C content increasing about 0.88 mol% for every 1 mol% increase in host G+C, with about 81 mol% of the variation being explained by this relationship (*r*^2^=0.81).

## Discussion

The data presented here demonstrate that there is a correlation between the length of a bacterial genome and its G+C content, particularly in the case of organisms with longer genome lengths. However, it is also clear that this alone is not enough to explain the complete variation in genome G+C content as evidenced by the results from the linear regression model. Therefore, it is clear that other factors need to be considered to explain the G+C content. Probably the most obvious of these would be the organism’s normal optimal temperature range [10, 11], as the physical property of having a high percentage of triple bonds (G+C rich) is more likely to prevent denaturing of double-stranded DNA than would be the case for those with a high percentage of double bonds (AT rich). However, other environmental factors also need to be considered as well [7–9], together with the physiological capabilities of the organism [8]. Moreover, the heteroskedasticity of the length versus percentage G+C plot suggests that multifactorial variables may be most important in terms of organisms with shorter genome lengths, arguing that the roles played by environmental factors in terms of influencing the G+C content of a bacterial genome will require meta-analytical approaches to elucidate the other key factors. It is also worth noting that to date there has been a bias towards sequencing genomes of organisms that are either medically or agriculturally important. It will be interesting to determine whether the patterns observed continue as more bacterial genome sequences become available from organisms that are not medically important or from those that lack agricultural significance.

In the case of the chromosomal analysis, the G+C content does not go above 75 mol% or below 13 mol%. In part, this may be a reflection of the restrictions of the genetic code, where encoding certain amino acids requires at least some usage of A/T or G/C, e.g. phenylalanine requires TTC or TTT as a codon (with G+C-rich organisms likely to favour TTC) and glycine requires GGN as a codon (with A+T-rich organisms likely to favour either GGA or GGT). In addition to this requirement of compliance to the genetic code, there may also be restrictions imposed whereby unusual or rare codons are incorporated into genes [19], with the possible effect of slowing down the rate of translation to allow correct protein folding to take place. Moreover, there is evidence to suggest that DNA replication in organisms with a higher G+C content is associated with variants in the presence of DNA polymerases present such as *polC* being used, in addition to the number of and types of variants of the *dnaE* gene [20], as evidenced by organisms such as *Pseudomonas putida* [21].

Plasmids can be considered as genetic components of the bacterial cell and it is not surprising that their G+C content is correlated to that of their host. This observation has previously been discussed by Campbell and colleagues [22], where a substantial similarity in genomic signatures between prokaryotes and their plasmids was reported, although more recently Rocha and Danchin [23] reported that genetic elements that can be considered as ‘intracellular pathogens’, such as plasmids, phages and insertion sequences, have a tendency to have a lower G+C content than their host organism. However, this conclusion was drawn from a much smaller dataset relative to the current work. Moreover, with the potential benefits associated with some genes on plasmids, it makes sense to see a similarity in terms of G+C content for plasmid-borne genes that rely on the transcriptional and translational factors of the host organism (e.g. the encoding of specific tRNA molecules by the bacterial host). It has also been proposed that similarity in G+C content acts as a way of allowing the bacterial cell to discriminate between compatible and non-compatible DNA [24], although factors such as methylation patterns ensure that this is not as simple a mechanism as relying on the G+C content alone.

Moreover, the increasing number of examples of lateral gene transfer, or horizontal gene transfer, shows that inter-species transfer of genes is more commonplace than first imagined. While there are other means of moving DNA from one organism to another, using plasmids as a vector for this transfer is regarded as one of the most important. This is true for both inter-species conjugation of plasmids or transformational uptake of plasmids that have been released into the ecosystem by an alien species. Therefore, although the plasmids described are known to have been isolated from a particular bacterial species, it is impossible to determine when this plasmid first became part of the bacterial cell, and also what previous organism(s) may have acted as the prior host(s). As above, it will be interesting to put this into context based on both bacterial and plasmid sequence data when sequences from additional organisms become available.

Conversely phages can be regarded as being true parasites of the cells depending on the host organism for expression of their genes, without the potential associated benefit of factors such as antibiotic-resistance genes. However, this in turn also places a dependence on them to maintain a G+C pattern similar to that seen in the organisms they infect. As mentioned above, there have been reports to suggest that intracellular pathogens may have a G+C content lower than their host organism [23], and we also find this to be the case in the current analysis of phages, based on a much larger dataset than was used previously. The evolutionary explanation for this is unclear, although reducing the phage’s metabolic burden via reduced pyrimidine synthesis has been proposed (e.g. [23, 25]).

In terms of phage genome analysis, the site of any incorporation into the bacterial genome (e.g. as part of any lysogenic cycle) could also influence the G+C content of the phage genome. This would be in keeping with reports of heterogeneity of G+C content across bacterial genomes [26], where sliding window analysis identified regions of intragenomic variation of G+C content within a single species.

In conclusion, using a considerably increased dataset relative to previous work, we propose that a simple linear regression between bacterial chromosome length and G+C content accounts for at least some of the relationship. The same relationship is also true for bacterial chromosome G+C content and plasmid G+C content, although phages tend to have a lower G+C content than their hosts. However, in all cases there are other factors involved, although the true extent of each of these factors remains unclear, arguing for additional analyses via techniques such as principal component analysis or multiple regression analysis on data regarding the ecosystems from which organisms have been isolated.

## Data bibliography

1. Almpanis A, Swain M, Gatherer D, McEwan N. Jupyter notebooks, https://github.com/atolgrp/Microbial-G-C-Content (2018).
